# The treatment and rehabilitation of a critical COVID‐19 case in China

**DOI:** 10.1002/ccr3.3725

**Published:** 2020-12-29

**Authors:** Xiangde Zheng, Lin Tian, Qinggao Liu, Shilin Li, Fanwei Zeng, Fanxin Zeng

**Affiliations:** ^1^ Department of Clinical Research Center Dazhou Central Hospital Dazhou China; ^2^ Department of Disease Control and Prevention Health Commission of Dazhou City Dazhou China

**Keywords:** COVID‐19, functional rehabilitation, pulmonary lesions

## Abstract

The lung lesions of this COVID‐19 patient were slowly absorbed, and the clinical symptoms with shortness of breath were improved slowly in the recovery period.

## Graphical abstract

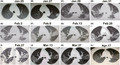

## 1 INTRODUCTION

We reported a critical case of COVID‐19, detail with his disease development, changes of lungs CT and rehabilitation for 11 weeks of follow‐up. The lung lesions were slowly absorbed, the texture of the pulmonary blood vessels were thickened, and the shortness of breath was improved significantly.

Since the outbreak of coronavirus disease‐19 (COVID‐19) in Wuhan City, Hubei Province, China, in December 2019, the epidemic spread rapidly all over the world. The data shared by the World Health Organization revealed that confirmed COVID‐19 cases 6 194 533, death cases 376 320 until 2 June 2020, in worldwide.^1^

Although much valuable experience have been accumulated during the diagnosis and treatment of COVID‐19, there is still much quandary that requires long‐term observation and understanding, such as multi‐time negative nucleic acid test results of confirmed cases, functional rehabilitation degree of critical patients, whether the pulmonary fibrosis could be absorbed fully and the period required for absorption.

We reported the diagnosis and treatment of a typical and critical patient of COVID‐19, as well as 3 months of follow‐up, for understanding the effects on health deeply.

## 2 CASE REPORT

**FIGURE 1:**
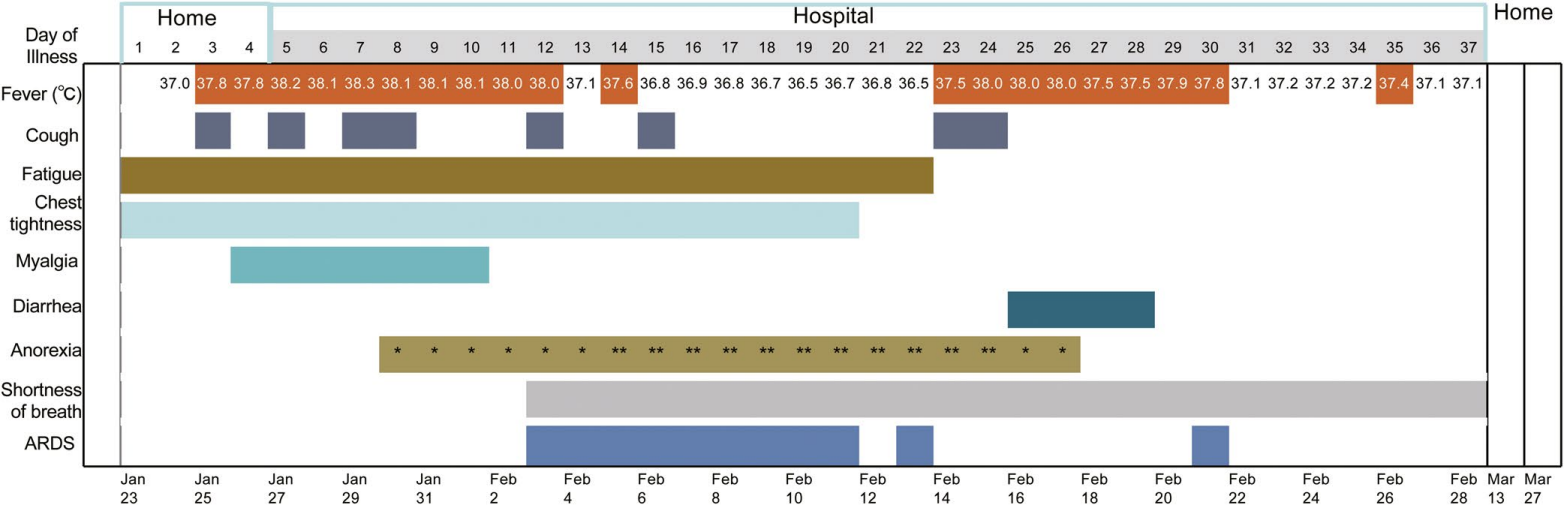
Symptoms and maximum body temperatures according to day of illness, January 23 to February 28, 2020

**FIGURE 2:**
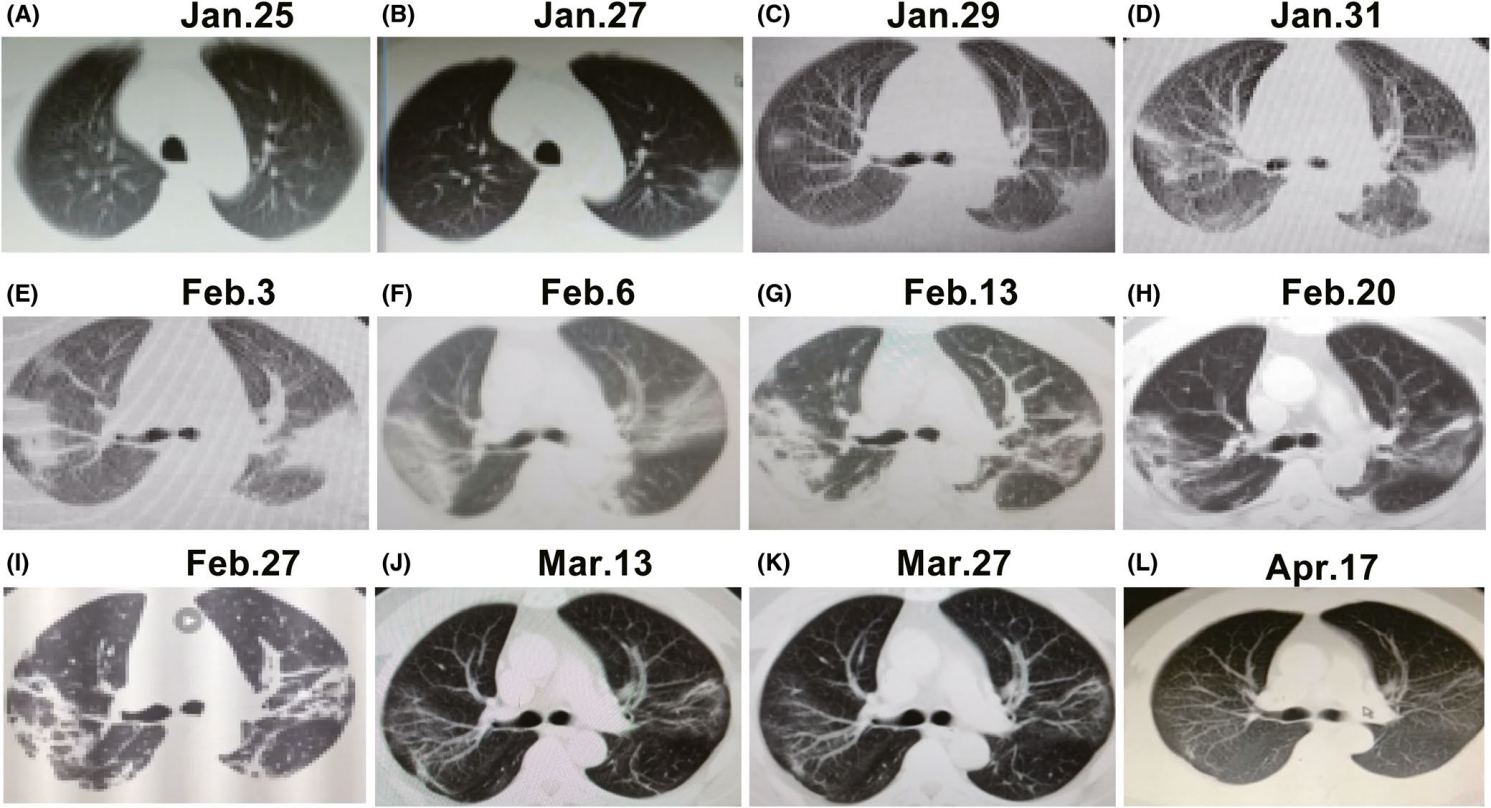
Changes of the successive CT images, January 25 to March 13, 2020. A, The both lungs were normal on illness day 3. B, The ground glass shadow was seen only in the posterior segment of the upper lobe of the left lung and near the subpleural has on illness day 5. C, Lamellar ground glass density shadows were seen in the posterior segment of the upper lobe in the left lung near the pleura, and patchy ground glass density shadows was scattered in the upper lobe of the right lung and a few cord shadows was seen in the lower lobe of both lungs on illness day 7. D, The ground glass density shadow was seen of left lung upper lobe posterior segment near the subpleural, partially consolidating; strip‐like, band‐like density increasing shadow were seen in the right upper lobe and the both lungs in the lower lobe, and the border was blurred on illness day 11. E, The two lungs continued to progress and partly consolidated; the upper and lower lobe of the right lung showed increased strip density and banded density, and the border was blurred on illness day 12. F, The two lungs began to absorb, and the consolidation density became shallower on illness day 16. G, The both lungs fibrosis changed after gradual absorption on illness day 22. H, The both lungs had smaller flake ground glass density shadows after gradual absorption on illness day 29. I, The both lungs had strip shadow after gradual absorption on illness day 36. J, Obvious absorption and a small amount of strip shadow was seen in both lungs on discharge day 14. K, A small amount of strip shadow was seen in both lungs 4 wk after discharge. L, The lesion was well absorbed and no pulmonary artery widening was seen 7 wk after discharge

The 45‐year‐old male employee from a company in Wuhan, the epidemic center, returned to his hometown Dazhou in Sichuan Province on January 23, 2020. On the same day, he began to have chest tightness and fatigue. On January 25, 2020, he developed fever (37.8°C) (Figure 1), but with normal hematological index and chest computer tomography (CT) scan (Figure 2A).

However, on illness day 5 (January 27, 2020), his symptoms became severe. He had chest tightness, lower limb weakness, accompanied by cough and fever. And his lungs CT showed ground glass near the pleura in the posterior segment of the upper lobe of the left lung. He was admitted to the local country hospital for isolation and treatment, as a suspected case of COVID‐19. On admission, the medical examination findings revealed a body temperature of 36.5°C, oxygen saturation (SpO_2_) of 97% while breathing ambient air, partial pressure of oxygen (PO_2_) 78.5 mm Hg (inhaled air), oxygenation index 373 mm Hg, white blood cells (WBC) 6.3 × 10^9^/L, neutrophil proportion 71.1%, lymphocyte count 1.25 × 10^9^/L (Figure S1), together with normal sensitive C‐reactive protein and blood biochemical index. The influenza A and B virus antigens and mycoplasma pneumoniae antibody IgG tests were negative. And then he was treated with oral ɑ‐interferon nebulization and lopinavir/ritonavir (Clitraz) as antiviral symptomatic supportive therapy.

And the 4 successive lungs CT images of the patient showed progressive enlargement of the left lung lesions, and a large invasion lesion of the right lung (Figure 2B‐E). Both lungs progressed from ground glass shadow and patch shadow to consolidation and strip shadow.

On illness days 15 (February 6, 2020), the patient progressed to critical COVID‐19 with moderate acute respiratory distress syndrome (ARDS), and was transferred to intensive care unit (ICU). His physical examination showed body temperature 36.7°C, arterial blood gas PCO_2_ 34.2 mm Hg, PO_2_ 53 mm Hg, oxygenation index 118 mm Hg (mask oxygen 6 L/min), highest WBC counts (13.8 × 10^9^/L) and highest neutrophil counts (12.53 × 10^9^/L), lowest lymphocyte counts (0.63 × 10^9^/L) (Figure S1). Meanwhile, the lungs CT showed large areas of patch, cord, and consolidation in both lungs (Figure 2F). Since his admission, he has received noninvasive positive‐pressure mechanical ventilation, combined with prone ventilation, anti‐infection, antiviral, dialectical treatment of traditional Chinese medicine and symptomatic supportive treatment. He was taken off the ventilator on February 12, 2020. His physical examination showed SpO_2_ 96.7%, PCO_2_ 42.8 mm Hg, PO_2_ 87.7 mm Hg, oxygenation index 302 mm Hg, WBC 7.56 × 10^9^/L, neutrophil proportion 74.2%, lymphocyte count 1.25 × 10^9^/L (Figure S1).

He was transferred to a general isolation ward from the ICU on illness day 22 (February 13, 2020). Three more rechecks of CT showed multiple patchy shadows and fibrotic changes in both lungs, from February 13 to February 27, 2020 (Figure 2G‐I).

On February 24, 2020, the patient underwent the 9th bronchoscopic alveolar lavage fluid SARS‐CoV‐2 nucleic acid test and SARS‐CoV‐2 next generation sequencing (NGS) test with both negative outcomes. Although the outcome of total antibodies of COVID‐19 test was positive. He received a total of 9 nucleic acid tests, and the results were all negative (Table 1).

**TABLE 1:** Results of SARS‐COV‐2 nucleic acid test, antibody test and NGS test^a^

Test	Illness day 5	Illness day 7	Illness day 9	Illness day 12	Illness day 13	Illness day 15	Illness day 16	Illness day 17	Illness day 27	Illness day 33 ^b^	Discharge day 14	Discharge day 28
Nucleic acid	−	−	−	−	−	−	−	−	NT	−	−	−
NGS	NT	NT	NT	NT	NT	NT	NT	NT	NT	No microorganisms	NT	NT
Total antibody	NT	NT	NT	NT	NT	NT	NT	NT	++	++	+	++
IgM	NT	NT	NT	NT	NT	NT	NT	NT	NT	NT	++	++
IgG	NT	NT	NT	NT	NT	NT	NT	NT	NT	NT	++	++

^a^ NT denotes not tested. − denotes negative; + denotes weakly positive; ++ denotes Positive.

^b^ Denotes the alveolar lavage fluid SARS‐CoV‐2 nucleic acid test was negative on illness day 33. The throat swabs and stool SARS‐CoV‐2 nucleic acid tests all were negative on discharge day 14.

## 3 FOLLOW‐UP

After discharged, the patient felt shortness of breath after the activity with large fibrous strips in both lungs. The focus of long‐term follow‐up was recovery of lung function and the absorption of pulmonary lesions.

The main follow‐up content included the COVID‐19 IgM and IgG antibodies tests, the change of lung imaging and lung function assessment. A modified version of the British medical research council respiratory distress scale (mMRC) was used to assess the degree of dyspnea. The higher mMRC score indicates more difficult breathing (ranges from 0 to 4).

The patient met the discharged standards on February 28, 2020, with shortness of breath after light activity (mMRC = 3), basically stable physical condition and disappeared clinical symptoms.

Two weeks after discharge (March 13, 2020), the patient did not fell other discomfort, apart from shortness of breath after activity (mMRC = 3), with normal hematological index. CT scan of both lungs showed striated shadow with obvious absorption (Figure 2J). Both IgM (60.5 AU/mL) and IgG (54.2 AU/mL) antibodies tests of COVID‐19 were positive and the SARS ‐CoV‐2 nucleic acid tests of throat swabs and stool both were negative. Combined with the positive COVID‐19 antibodies tests and the typical clinical symptoms, he was revised to confirmed case according to the 7th edition of the New Coronavirus Pneumonia Diagnosis and Treatment Plan.

Four weeks after discharge (On March 27, 2020), the second followed‐up result showed only slight shortness of breath after activity (mMRC = 2). The CT scan of both lungs still showed striated shadow, with no significant change compared with the CT results on March 13, 2020 (Figure 2K). IgM (40.0 AU/mL) and IgG (72.2 AU/mL) antibodies tests of COVID‐19 were positive and the SARS‐CoV‐2 nucleic acid tests of throat swabs and stool both were negative.

Seven weeks after discharge (On April 17, 2020), the patient still felt shortness of breath after the activity (mMRC = 2) and started to work with a normal life. The CT result showed that the lesions of both lungs were significantly absorbed compared with the CT on March 27, 2020, the blood vessels in the lesion area became thicker, and no pulmonary artery widening was observed (Figure 2L).

Eleven weeks after discharge (On May 17, 2020), the patient still felt slight shortness of breath after activity (mMRC = 1), and with occasional chest tightness.

## 4 DISCUSSION

It is the deepening of the understanding of COVID‐19, the accumulation of clinical diagnosis and treatment experience, and the timely update of the 7 editions of the New Coronavirus Pneumonia Diagnosis and Treatment Plan of the National Health and Health Committee of China,^2^ that make a huge contribution to the prevention and control of the epidemic. In spite of these clinical practice, some patients often have special manifestations, such as long incubation period, typical clinical symptoms with multiple times negative results of nucleic acid test, or returning positive of nucleic acid test after discharging,^3^, ^4^, ^5^ which increase confusion to clinical diagnosis and treatment.

The patient of our case had a clear history of exposure to Wuhan, and clinical symptoms of COVID‐19 (chest tightness, fatigue, fever, and lung subpleural ground glass shadow). His total 9 times of throat swab nucleic acid tests were negative, and the nucleic acid test and NGS test of alveolar lavage fluid were negative. He was finally confirmed COVID‐19 based on his clinical symptoms, physical signs and typical CT images, combining with the positive outcomes of COVID‐19 antibodies tests during hospitalization and rehabilitation.

When the patient at the critical stage, it was observed that the improvement on oxygenation was still slow even if treated with ventilator supportive. This phenomenon was confirmed by the pathological anatomy of the first COVID‐19 death patient in China.^6^ The pathological manifestations of lung tissue were diffuse alveolar injury with exudation of cell fiber mucus, together with alveolar epithelial cells shedding, pulmonary edema, and pulmonary clear membrane formation, which causes oxygen mass disorders.

The patient's lymphocyte count was normal at the time of the onset, began to decrease on illness day 12, and decreased to the trough on illness day 16. But the changes tendency of the WBC and neutrophils were opposite with lymphocyte, forming an increasing wave. At this period, the lung exudation and consolidation lesions were the most prominent, and the absorption was slow in the later. This manifestation may be related to combined nosocomial infections after the body's immune function continuously impaired, which aggravated the acute respiratory distress syndrome (ARDS). The manifestation of this clinical symptom aggravation was basically consistent with the change of CT imaging of the lungs and may be the main reason for the patient was transfer to the ICU on February 6, 2020.

Although the nucleic acid tests of the patient were negative, his positive COVID‐19 antibody tests after discharge and the gradually increased titer indicated the fact that the COVID‐19 infection existed in the early stage. The false‐negative nucleic acid test and false‐positive antibodies test in COVID‐19 are the practical problems for diagnosis. The reason may be related to sample collection and storage, viral infection sites, RNA extraction, and quality of nucleic acid detection kits.^7^, ^8^ The false negative or false positive for detection of COVID‐19 antibody was also existent, which was not suitable for general population screening for returning to work and school, nor suitable for epidemiological investigation in low‐prevalence areas. COVID‐19 IgM and IgG antibody tests should be applied as a supplement or conjunction with nucleic acid tests for suspected cases.^9^

It was observed that the absorption of pulmonary lesions in the patient was slower in the early stage than the later stage. Whether the patients could fully absorb the lung lesions need to further observed. At the same time, it is necessary to pay attention to whether pulmonary arterial hypertension will occur in the later stage, as well as the changing trend of titer of COVID‐19 antibody in the future.

## 5 CONCLUSION

It took long time to recover from lung injury for the COVID‐19 patient. The lung lesions were slowly absorbed and the shortness of breath was improved slowly in the recovery period, and the texture of the pulmonary blood vessels thickened.

## CONFLICT OF INTEREST

All authors declare that they have no known competing financial interests or personal relationships that could have appeared to influence the work reported in this paper.

## AUTHOR CONTRIBUTIONS

XZ: served as attending physician for the case and primary author of the case report. LT and QL: served as members of medical team for the case. FanwZ: served as a member of medical team and editor of the case report. FanxZ: served as primary editor of the case report. SL: served as an editor of the case report.

## ETHICAL APPROVAL

Informed consent was obtained from the patient for publication of this case report and accompanying image.

## Supporting information

Fig S1

## Data Availability

The data that support the finding of this study are available from the corresponding author upon reasonable request.
