# Supplementary material for: The treatment and rehabilitation of a critical COVID‐19 case in China
Source: Clin Case Rep. 2020 Dec 29;9(2):990–4. doi: 10.1002/ccr3.3725 (PMC7869341; doi:10.1002/ccr3.3725)
Supplement: Supplementary file 1 — Fig S1 [file CCR3-9-990-s001.doc]

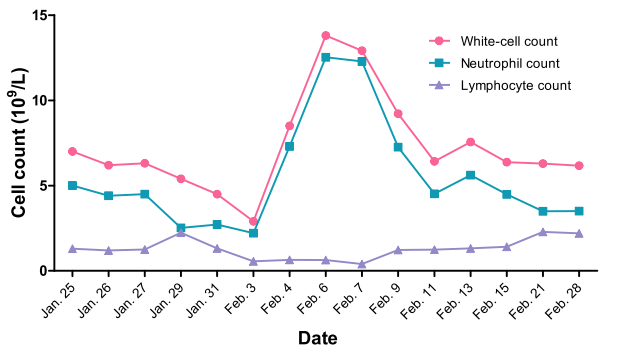


Figure S1. Changes tendency of the white-cell count, lymphocytes cell count and neutrophils cell count in patients.
